# Epidemiology for Undergraduates

**Published:** 2009-07

**Authors:** Sanjay Zodpey

**Affiliations:** Director, Public Health Education, Public Health Foundation of India, New Delhi, India. E-mail: sanjay.zodpey@phfi.org

The primary objective of the book is to introduce the subject of epidemiology to medical graduates in a simple and lucid way. The idea is to introduce the fundamentals and applications of epidemiology in such a manner that they are thought provoking and give students an opportunity to learn by themselves. The book provides comprehensive compilation of epidemiology from descriptive and analytic methods to statistical techniques for analysis of those designs and methodologies for measurement. It incorporates the concepts of quantitative and qualitative research methods, decision making of diagnostic tests, screening as well as surveillance and encourages the disciple to endeavor to reach domains of higher learning by inspiring interest in the subject.

The book has been organized systematically into five modules, each sub-divided into various chapters. The modules deal with traditional and emerging themes in epidemiology like epidemiologic methods, measurements in epidemiology, statistics in epidemiology, practical epidemiology and applied epidemiology. The chapters are succinctly written with specific objectives and exercises, replete with numerous examples which are very useful and helpful for learning the concepts and methods of epidemiology in a step wise fashion.

This book can be an excellent primer for medical graduates for epidemiology. It has all the necessary ingredients to stimulate a graduate's interest in epidemiology as well as enough scientific merit to be recommended as essential reading for undergraduate teaching.

The layout of the content in modules and chapter with specific learning objectives is commendable. However, the learning objectives fall short in the context of hierarchy of knowledge and so is the content. The nomenclature of the modules/selection of chapters within the module could have been better: surveillance and investigation could have been under field epidemiology; the module on applied epidemiology could have introduced emerging concepts in infectious disease epidemiology, non-communicable disease epidemiology, genetic epidemiology, etc. The book in its present form is still praise-worthy for presenting the subject in a different perspective and has all the potential to stimulate and kindle the epidemiologist in the medical graduate and hence is more than welcome.

